# AI algorithms for accurate prediction of osteoporotic fractures in patients with diabetes: an up-to-date review

**DOI:** 10.1186/s13018-023-04446-5

**Published:** 2023-12-12

**Authors:** Zeting Li, Wen Zhao, Xiahong Lin, Fangping Li

**Affiliations:** 1 Department of Endocrinology, The Seventh Affiliated Hospital, Sun Yat-sen University, Shenzhen, China; 2 The Reproductive Center, The First Affiliated Hospital, Sun Yat-sen University, Guangzhou, China

**Keywords:** Osteoporotic fracture, Artificial intelligence, Fracture prediction, Diabetes

## Abstract

Osteoporotic fractures impose a substantial burden on patients with diabetes due to their unique characteristics in bone metabolism, limiting the efficacy of conventional fracture prediction tools. Artificial intelligence (AI) algorithms have shown great promise in predicting osteoporotic fractures. This review aims to evaluate the application of traditional fracture prediction tools (FRAX, QFracture, and Garvan FRC) in patients with diabetes and osteoporosis, review AI-based fracture prediction achievements, and assess the potential efficiency of AI algorithms in this population. This comprehensive literature search was conducted in Pubmed and Web of Science. We found that conventional prediction tools exhibit limited accuracy in predicting fractures in patients with diabetes and osteoporosis due to their distinct bone metabolism characteristics. Conversely, AI algorithms show remarkable potential in enhancing predictive precision and improving patient outcomes. However, the utilization of AI algorithms for predicting osteoporotic fractures in diabetic patients is still in its nascent phase, further research is required to validate their efficacy and assess the potential advantages of their application in clinical practice.

## Introduction

Osteoporotic fractures (OF) remains a prevalent clinical disorder that severely impacts patients' quality of life, leading to hospitalization, disability, and even mortality [1]. The diagnosis of osteoporosis is established when the bone mineral density (BMD) value falls below − 2.5 standard deviations (T-score − 2.5) [2]. Latest globalized data has indicated that the prevalence of osteoporosis and osteopenia were 19.7% and 40.4%, respectively [3]. OFs is the most common complication of osteoporosis, including hip fracture (HF), vertebral fracture (VF), wrist fractures (WF), and distal radius fracture (DRF). An estimated 9 million cases of OF were reported worldwide in 2000, including 1.6 million HF, 1.7 million WF, and 1.4 million VF [4]. Notably, the mortality rate after HF may be as high as 20% [5]. Given the substantial morbidity and serious consequences associated with OF, this condition imposes a significant economic burden on society that cannot be ignored. In 2000, osteoporosis in the UK was accountable for a financial burden of 1.8 billion pounds, and this figure is estimated to rise to 2.2 billion pounds by 2025 [6]. Similarly, China is projected to experience 5.99 million osteoporotic fractures annually, with an annual cost of 25.43 billion US dollars by 2050 [7]. Aging remains the primary etiological factor that underlies osteoporosis, while secondary causes include chronic kidney disease, diabetes, thyroid disease, treatment of glucocorticoids, proton pump inhibitors, antiepileptic drugs, and selective serotonin reuptake inhibitors [8]. With the global trend of population aging, OF will undoubtedly become more prevalent [9]. Thereby emphasizing the need for accurate risk assessment to minimize the substantial socioeconomic costs associated with this condition.

In addition to the rising incidence of osteoporosis, diabetes is also a growing public health concern. Interestingly, although patients with type 1 diabetes mellitus (T1DM) tend to have lower BMD, patients with type 2 diabetes mellitus (T2DM) often exhibit higher BMD [10]. Despite these differences in BMD, both T1DM and T2DM increase the risk of fracture [11]. FRAX, QFracture algorithm and the Garvan Fracture Risk Calculator (Garvan FRC) are the most common fracture risk prediction tools internationally. However, these tools fail to predict the fracture risk of patients with diabetes accurately [12]. Specifically, FRAX and Garvan FRC underestimate fracture risk in patients with T2DM by failing to incorporate T2DM as an independent predictor. On the other hand, although QFracture includes both T1DM and T2DM as independent predictors, its performance has not been validated in diabetic populations [12]. As such, there is a pressing need for more accurate fracture risk prediction tools for individuals with diabetes.

This comprehensive review aims to provide insights into the unique features of bone metabolism and fracture risk in patients with osteoporosis and diabetes mellitus. Specifically, we critically analyze the limitations of currently available osteoporosis prediction tools for diabetic populations and explore the potential of artificial intelligence (AI) in enhancing the accuracy of fracture prediction. Our findings highlight the urgent need for innovative approaches to personalized fracture risk assessment and management.

## Correlation of diabetes and osteoporotic fracture

Distinct pathophysiological mechanisms of T1DM and T2DM underlie the heightened fracture risk observed in patients with diabetes. The risk for fractures in patients with T1DM is six-fold higher than in the general population, primarily due to low BMD, alterations in bone quality, microarchitecture, and impaired bone turnover state [13]. Non-osseous factors such as recurrent hypoglycemic episodes, peripheral neuropathy, autonomic neuropathy, retinopathy, and low body weight further increase the risk of falls in this population [14]. Although the decrease of BMD is not significant, T2DM patients still have a higher risk of fracture than non-diabetes patients [13, 15]. A meta-analysis comprising 54 clinical studies conducted in China has demonstrated that the prevalence of osteoporosis in patients with type 2 diabetes is significantly higher (37.8%) than the overall prevalence of osteoporosis (27.96%) [16]. In sharp contrast to the effect of secondary OF in T1DM, T2DM patients tend to present with higher BMD [17]. Alteration in bone microarchitecture that result in poor bone quality may account for the increased risk of fracture in T2DM patients. Studies have shown that trabecular bone score (TBS) in patients of T2DM and pre-diabetes stage is significantly lower than that in non-type 2 diabetes patients [18]. The changes of bone microstructure in T2DM with OF were characterized by higher endocortical bone surface, intracortical pore volume and greater relative porosity at the distal tibia and ultra-distal radius [19]. In addition to the risk factors above mentioned, chronic hyperglycemia, tissue-specific accumulation of advanced glycation end-products (AGE), changes in vitamin D homeostasis, diabetes microvascular disease, and insulin pharmacotherapy [20], which are common to the two types of diabetes, have adverse effects on the bone health of diabetes patients. In view of the large population base of diabetes patients, the unique bone metabolism characteristics, and higher prevalence of osteoporosis, OF should not be neglected as a complication of diabetes.

## Prediction effect of traditional prediction tools on fracture in diabetes patients

At present, there are various risk assessment tools for OF. The most recommended risk assessment tool is the FRAX® fracture risk assessment tool [21], followed by the QFracture algorithm [22] and the Garvan Fracture Risk Calculator (Garvan FRC) [23].

FRAX (https://www.sheffield.ac.uk/FRAX/) is a computer-based algorithm that calculates the fracture risk in the next 10 years for people aged 40–90. Since it was developed in 2008, FRAX has been an open access tool provided convenience for clinicians all over the world. The algorithm incorporates independent variables included age, weight (kg), height (cm), previous fracture history, parent hip fracture history, smoking history, glucocorticoid use history, rheumatoid arthritis history, secondary osteoporosis history, alcohol intake history, and femoral neck BMD value. Obviously, race, diet, geographical factors that vary from regions to regions are not included. The reason that FRAX is recommended by most osteoporosis related guidelines or consensus all over the world is that FRAX has been calibrated to countries or regions where the epidemiology of fracture and death is known (currently 64 countries) [21]. FRAX regards T1DM but not T2DM as a cause of secondary osteoporosis, and the current FRAX algorithm does not acquire T2DM input, which may be the reason why FRAX has insufficient ability to predict fracture in patients with diabetes [24]. As for T1DM, scientists haven made efforts to use FRAX algorithm to predict fracture in patients with T1DM without BMD value in a clinical cohort of 346 patients with T1DM and 411 controls, and concluded that the FRAX without BMD exerted good prediction efficiency in detecting patients with T1DM at risk of major osteoporotic fracture (MOF) [25]. However, it has been recognized that FRAX underestimates the fracture risk of individuals with T2DM when applied to populations with equivalent BMD and T score [26]. In 2012, a clinical study including 3518 diabetes patients and 36,085 non diabetes patients showed that FRAX underestimated the risk of MOF and HF observed in diabetes patients after adjusting for competitive mortality [27]. Furthermore, a cohort study of 49,098 non-diabetic women and 8840 women with diabetes in 2016 showed that FRAX underestimated the risk of HF in diabetes patients, while in diabetes patients with diabetes for more than 10 years, FRAX underestimated the risk of MOF [28]. To improve the performance of FRAX for T2DM, four feasible methods have been proposed [29]: (1) including the rheumatoid arthritis (RA) input to FRAX; (2) making a TBS adjustment to FRAX; (3) reducing the femoral neck T-score input to FRAX by 0.5 SD; and (4) increasing the age input to FRAX by 10 years. However, there has not been a method that is optimal in all cases.

QFracture (https://qfracture.org/) was developed by Julia Hippisley Cox and Carol Coupland based on data from the United Kingdom to estimate1 to 10-year risk of MOF and HF in people aged 30–99 without BMD measurement. It is characterized by no need for imaging or laboratory examination data. The variables in this algorithm included body mass index (BMI), age, gender, ethnicity, smoking status, alcohol status, history of diabetes, parent’s history of osteoporosis/hip fracture, residence history of nursing or care home, history of wrist spine hip or shoulder fracture, history of falls, dementia, cancer, asthma or chronic obstructive pulmonary disease (COPD), heart attack, angina, stroke or transient ischemic attack (TIA), chronic liver disease, chronic kidney disease (stage 4 or 5), Parkinson's disease, rheumatoid arthritis or systemic lupus erythematosus (SLE), malabsorption such as Crohn's disease, ulcerative colitis, coeliac disease, steatorrhea or blind loop syndrome, endocrine problems such as thyrotoxicosis, hyperparathyroidism, Cushing's syndrome, epilepsy or treatment of anticonvulsants, antidepressants, treatment of steroid tablets regularly, and estrogen replacement therapy. Compared with FRAX, the advantage of QFracture algorithm applied in the prediction of osteoporotic fracture in diabetics is that it directly includes the presence or absence of diabetes as its calculation variable. However, there are currently no studies specifically evaluating the performance of QFacture prediction of OF in patients with diabetes. Despite the disadvantages including not taking BMD as an input variable, ignoring mortality as a competitive risk, and only calibrated for use in populations in UK, its clinical use is still recognized. The Fremantle Diabetes Study Phase I (FDS1) in 2019 [30] proposed a simple HF risk prediction tool which took QFacture as comparison to evaluate the prediction efficiency of MOF. QFracture demonstrated excellent discrimination, calibration, and accuracy. During 10 years of follow-up, 48 (3.94%) out of the 1219 members of the FDS1 cohort with T2DM had an incident HF, and the predicted risk by the QFracture hip fracture risk equation is 4.06% (49.5 cases).

Garvan FRC was developed based on the Dubbo Osteoporosis Epidemiology Study (DOES) cohort study in Australia in 2007 [31]. Garvan FRC incorporates four clinical risk factors including age, sex, number of previous fractures, and number of recent falls with BMD or weight (when BMD is not available) to estimate 5-year and 10-year risk of OF and HF [32]. Although diabetes is not incorporated in Garvan FRC’s risk prediction algorithm, history of recent falls may substitute for the increased risk of diabetes to a certain extent. A registry-based cohort study was performed in 2022 to estimate the performance of Garvan FRC in patients with diabetes [33]. Individuals aged 50–95 years consisting of 2618 women with and 14,064 without diabetes, and 636 men with and 2201 without diabetes were recruited and their 5-year fracture risk rate was calculated. Results showed that Garvan FRC provided similar fracture risk stratification in individuals with versus without diabetes, however, OF risk in women with diabetes was underestimated. Interestingly, after lowering the femoral neck T-score by 0.3 in women with diabetes for re-calculation, the effect of diabetes on OF and HF provided by Garvan FRC was largely attenuated.

As mentioned above, clinicians use of FRAX, QFracture, and Garvan FRC for estimation of fracture risk in an individual with diabetes should be aware of this limitation.

## Performance of AI in predicting osteoporotic fracture

Since the above three tools have limitation in predicting osteoporotic fracture in patients with diabetes, there is an urgent need to develop a convenient and accurate tool to predict the risk of OF in patients with diabetes. In recent years, the rapid development of artificial intelligence has brought a feasible answer.

Artificial intelligence (AI) technology has been increasingly utilized in the field of osteoporosis, primarily through machine learning (ML) methodologies. ML is to apply specific traits to identify patterns that can be used to analyze a particular situation [34]. Algorithms of ML are typically classified into three typical types: supervised learning, unsupervised learning, and semi supervised learning. The typical supervised learning algorithms include linear regression, logistic regression, neural network, decision tree, random forest, support vector machine, least absolute shrinkage and section operator, ensemble learning, and deep learning, etc. Unsupervised learning algorithms include K-means clustering, principal component analysis, support vector domain description, and local outlier factor, etc. Semi supervised learning is an algorithm that combines supervised learning and unsupervised learning but rarely used in the medical field. Supervised learning is frequently used to estimate risk. The area under the receiver operating characteristic curve (AUROC) and precision-recall curve (PRC) are comparatively common patterns to demonstrate the performance of AI algorithms [35]. These powerful algorithms allow for the identification of complex relationships and patterns within large datasets, offering great potential in advancing our understanding and management of osteoporosis.

To assess the current predictive value of AI in estimating osteoporotic fracture risk, a systematic literature search was conducted across multiple electronic databases, including PubMed and Web of Science. Studies published till March 2023 were included. A restriction for English language has been applied. Searches relevant to the use of AI to predict osteoporotic fracture were made using the search terms (“Fracture” OR “Fragility Fracture” OR “Osteoporotic Fracture”) AND (“Artificial Intelligence” OR “Deep Learning” OR “Machine Learning”) AND “Osteoporosis”. Records identified from searches were screened by the Li Zeting and Zhao Wen.

**Fig. 1:**
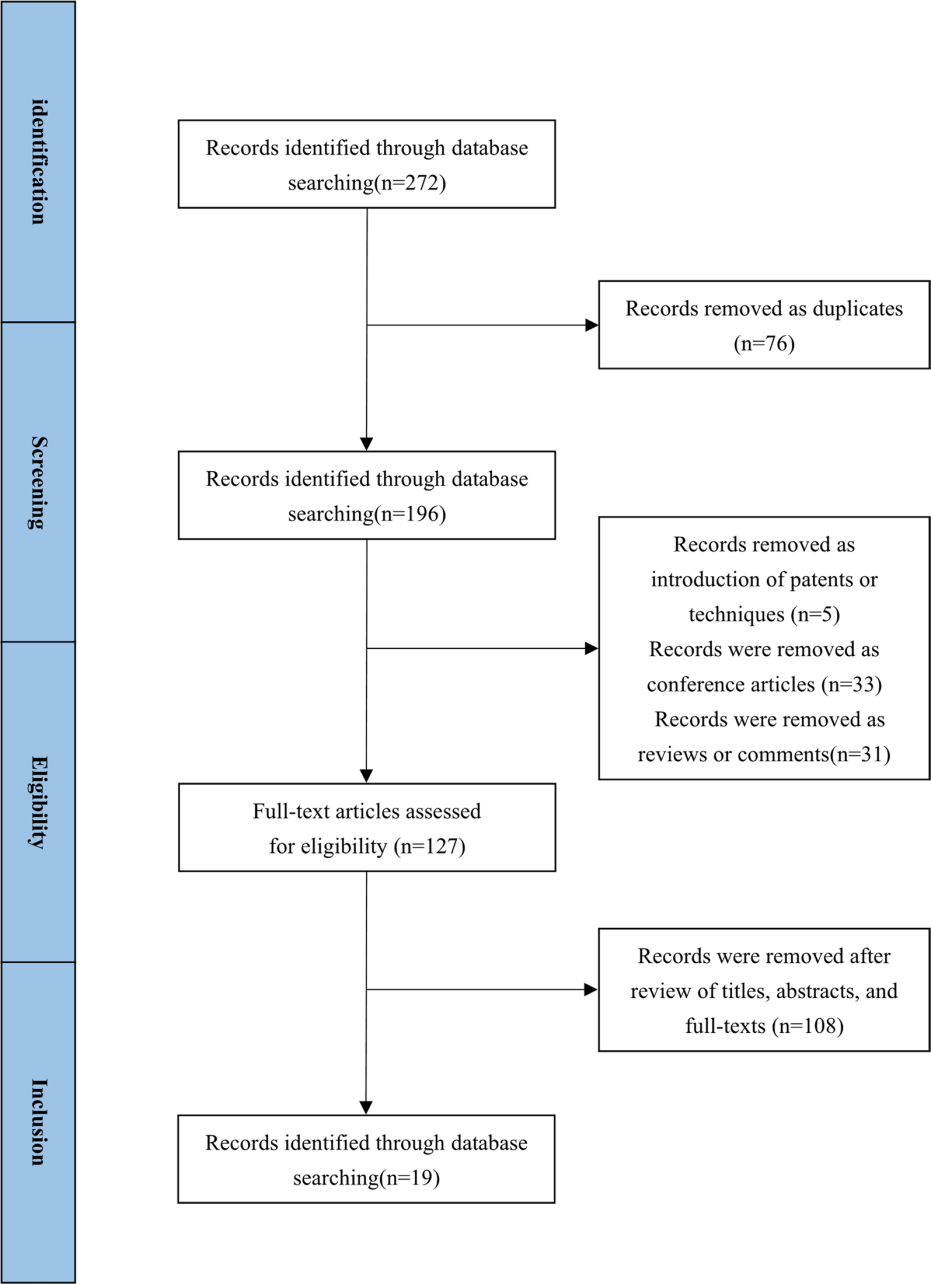
The study selection flow diagram

The study selection flow diagram is presented in Fig. 1. 272 literature results were identified by the search stragety.76 records were removed as duplicates. 5 records were removed as introduction of patents or techniques. 33 records were removed as conference articles. 31 records were removed as reviews or comments. 108 records were removed after review of titles, abstracts, and full-texts, among which 16 were irrelevant to the theme, and 92 identified as AI on osteoporosis in other fields, including 2 about applications of AI on omics, 44 about applications of AI on prediction of BMD and screening and diagnosis of osteoporosis, 24 about applications of AI on diagnosis of fracture, 17 about applications of AI on assessment of bone quality or bone microstructure, and 5 about applications of AI on iatrogenic intervention related to osteoporosis and its prognosis. A total of 19 of these records [36–54] concerned with the applications of AI on prediction of fracture risks were considered eligible for inclusion in the current literature review as presented in Table 1.

**Table 1:** AI methods on osteoporotic fracture prediction

Author	Time	Country	Mortality	Subjects	AI algorithm	Train/validation/test set	Best result
Whittier et al. [36]	2022	Canada	HR-pQCT	5873 patients	Fuzzy c-means clustering	446 train and 5873 test	HR = 2.96
Shimizu et al. [37]	2022	Japan	Database	7033 patients	DT, Feature Selection and Relative Importance, ANN, and SVM	75% train and 25% test	AUC = 0.74
Kong et al. [38]	2022	Korea	Database	1595 participants	DeepSurv	1416/1595 train (fivefold CV), and 179/1595 test	C-index = 0.614
Dong et al. [39]	2022	USA	Database	4461 subjects and 15,524 spine radiographs	GoogLeNet	76.5% train, 8.5% validation, and 15% test	AUC-ROC = 0.99, AUC-ROC = 0.82, sensitivity = 59.8%, PPV = 91.2%, and F1 score = 0.72
Chen et al. [40]	2022	China	Database	14,419 patients	XGBoost combining MLP	80% train and 20% test	AUC = 0.9 (approximately), accuracy = 90.38%, and F1 score = 0.9037
Ulivieri et al. [41]	2021	Italy	Database	172 women	2 derivative algorithms of ANN	90/172 train and 82/172 test, then reverse to 82/172 train and 90/172 test	AUC = 0.896, accuracy = 82.93%, sensitivity = 82.14%, specificity = 83.72%
Nissinen et al. [42]	2021	Finland	DXA	2949 + 459 women and 115 men	Convolutional neural network (CNN)	2949/3523 train (tenfold CV), and 574/3523 test	AUC = 0.64, accuracy = 52.0%, sensitivity = 67.8%, specificity = 51.4%
de Vries et al. [43]	2021	Netherland	Database	7578 patients	CR, RSF and ANN-DeepSurv model	100% train and 100% test	C-index = 0.625
Wu et al. [44]	2020	USA	Database	5130 men	RF, NN, LR, and gradient boosting,	80% train (tenfold CV), and 20% test	AUC = 0.71, Accuracy = 0.88
Villamor et al. [45]	2020	Spain	Database	137 patients	SVM, RBF, LR, SNN, and RF	101/137 train (tenfold CV), and 36/137 test	Accuracy = 0.86
Galassi et al. [46]	2020	Spain	Database	137 patients	SVM, LR, DT, and RF	70% train (twofold CV), and 30% test	Accuracy over 87%, Specificity over 92%, and Sensitivity over 83%
Engels et al. [47]	2020	Germany	Database	288,086 individuals	SL, XGBoost, LR, RF, SVM and RUS	80% train (tenfold CV), 20% test	AUC = 0.72
Almog et al. [48]	2020	USA	Database	6,329,986 patients	Crystal Bone	50% train (threefold CV), 50% test	AUC = 0.81
Su et al. [49]	2019	USA	Database	5994 men	CARTs	tenfold CV	AUC = 0.726
Muehlematter et al. [50]	2019	Switzerland	CT	60 stable and 60 unstable vertebrae of 58 patients	MLP, ANN, RF, SVM, and naïve Bayesian classifier	2/3 train (tenfold CV), 1/3 test	AUC = 0.97
Ferizi et al. [51]	2019	USA	Database	92 women	linear models, SVM, DT, KNN, and EL	22/23 train (23-fold CV), 1/23 test	Specificity = 0.83(adjusted), Accuracy = 0.71(adjusted), Precision = 0.68, F1-score = 0.67(adjusted)
Kruse et al. [52]	2017	Denmark	Database	10,775 women	Standardized variable means, Euclidean distances, and Ward's D2 method of HAC	Not required	Nine (*k* = 9) clusters were identified
Kruse et al. [53]	2017	Denmark	Database	4722 women and 717 men	Classification Tree, BAT, BGLM, PLS, KNN, LogitBoost, BGAM, HDDA, RF, C5.0, CIT, LMT, SGB, QDA, LDA, BFDA, BMARS, NSC, SVMRW, NN, NNFE, XGB, CIRF, and AB	75% train (fivefold CV), 25% test	AUC = 0.92
Schuler et al. [54]	2010	Australia	CT	100	InShape model	Not presented	*R* = 0.91

*AI* artificial intelligence, *CT* computed tomography, *ML* machine learning, *CV* cross validation, *AUC* area under curve, *BAT* Bootstrap aggregated trees, *BGLM* Bayesian Generalized Linear Model, *PLS* Partial Least Squares, *KNN* k-Nearest Neighbours, *LogitBoost* Boosted Logistic Regression, *BGAM* Boosted Generalized Additive Model, *HDDA* High Dimensional Discriminant Analysis, *RF* Random Forest, *CIT* Conditional Inference Tree, *LMT* Logistic Model Trees, *SGB* Stochastic Gradient Boostin, *QDA* Quadratic Discriminant Analysis, *LDA* Linear Discriminant Analysis, *BFDA* Bagged Flexible Discriminant Analysis, *BMARS* Bagged Multivariate Adaptive Regression Splines, *NSC* Nearest Shrunken Centroids, *SVMRW* Support Vector Machines with Radial Weights, *NN* Neural Network, *NNFE* Neural Network with Feature Extraction, *XGB* eXtreme Gradient Boosting, *CIRF* Conditional Inference Random Forest, *AB* Adaptive Boosting, *HAC* hierarchical agglomerative clustering, *DT* decision tree, *EL* ensemble learning, *MLP* multi-layer perceptron, *ANN* artificial neural networks, *SVM* support vector machine, *CARTs* classification and regression trees, *SL* Superlearner, *XGBoost* extreme gradient boosting, *LR* logistic regression, *RUS* random under sampling, *RBF* radial basis function, *SNN* Shallow Neural Networks, *GB* gradient boosting, *CR* cox regression, *RSF* random survival forests, *DXA* dual X-ray absorptiometry

According to the presented data, most researches (18/19) of application of AI on prediction of fracture risks showed up after 2017, which is related to the vigorous and rapid development of AI algorithm in recent years. Researchers from various localities have gained universally acknowledged achievements in this domain, especially researchers from North America [36, 39, 44, 48, 49, 51], Europe [41–43, 45–47, 50, 52, 53], and East Asia [37, 38, 40]. As for the application of algorithm patterns, supervised learning [41–51, 53, 54] is the most common pattern of risk prediction algorithm, and there are also reports on the application of unsupervised [36, 52] learning methods to define high-risk groups of OFs. Most of the existing studies are based on the existing cohort researches. In the field of fracture risk prediction, the application of AI is mainly divided into three types, among which the most common type is to improve the efficiency of fracture risk prediction by to establishing a new method or by applying the existing AI algorithm to the field of fracture risk prediction [38–40, 42–44, 46–49, 53, 54]. The following is to improve the prediction efficiency of the original ML prediction model or traditional prediction method by incorporating innovative imaging data or clinical characteristics [36, 37, 39, 41, 45, 50, 51]. Last but not least, to define the high-risk group [36, 52] through ML algorithm, and then calculate its fracture prediction risk.

The prediction efficiency of AI fracture prediction model is the most concerned in clinic. As shown in Table 1, most ML model excellent prediction efficiency, with AUC range from 0.74 to 0.99. Kruse et al. [53] made the greatest efforts in the application of the ML algorithm, that 24 ML algorithms were applied in their research to predict 5-year hip fracture and 10-year hip fracture for a population of 4722 women and 717 men, including Classification Tree, Bootstrap aggregated trees, Bayesian Generalized Linear Model, Partial Least Squares, k-Nearest Neighbours (kNN), Boosted Logistic Regression, Boosted Generalized Additive Model, High Dimensional Discriminant Analysis, Random Forest (RF), Conditional Inference Tree, Logistic Model Trees, Stochastic Gradient Boostin, Quadratic Discriminant Analysis, Linear Discriminant Analysis, Bagged Flexible Discriminant Analysis (BFDA), Bagged Multivariate Adaptive Regression Splines, Nearest Shrunken Centroids, Support Vector Machines with Radial Weights, Neural Network (NN), Neural Network with Feature Extraction, eXtreme Gradient Boosting (XGB), Conditional Inference Random Forest and Adaptive Boosting. Predictive performance and calibrated probabilities were good as AUC > 0.9 in ML algorithms such as XGB, RF, and BFDA, and the best performance is the BFDA in the female group with an AUC value of 0.91. These results surpassed the most widely used modified FRAX tools performance achieved by Lundin et al. (AUC = 0.73 [0.64;0.81]) [55]and that in the Spanish FRIDEX cohort (AUC = 0.88 [0.82; 0.95]) [56].

Adding new parameters to the existing ML model may obtain potentially beneficial effects. Dong et al. [39] modified GoogLeNet, a neural network algorithm, to 4461 subjects and 15,524 spine radiographs for osteoporotic compression fractures by combing radiographs Genant semiquantitative system, then achieved an AUC-ROC of 0.99 and an area under the precision-recall curve (AUC-PR) of 0.82, respectively. Meanwhile, a sensitivity of 59.8%, a specificity of 99.9%, a positive predictive value (PPV) of 91.2%, an F1 score of 0.72, and an accuracy of 99.5% were yielded.

In addition to improve the prediction efficiency, to identify the characteristics of high fracture risk population in the early stage is also of great clinical significance. Whittier et al. [36] classified a multinational cohort (*n* = 5873), into three phenotypes by fuzzy c-means clustering, an unsupervised ML method, with high-resolution peripheral quantitative computed tomography (HR-pQCT) data. These phenotypes were identified by their anatomically different characteristics in bone microarchitecture, and associated with stratified risk of osteoporotic fracture. Furthermore, within each phenotype, unique bone imaging biomarkers were associated with within-phenotype fracture risk. This fracture prediction model that predicted fracture risk through unsupervised learning method combined with imaging examination rather than other cumbersome laboratory examination results or clinical characteristics facilitated clinical workflow.

A growing body of research has compared the efficacy of various ML methods in fracture risk prediction. In general, newer ML techniques, such as random forest (RF) and neural networks (NN), have demonstrated superior performance compared to traditional approaches like logistic regression (LR). Furthermore, it is expected that deep learning (DL) methods will yield even better results in this domain. For instance, Wu et al. [44] established a prediction model of male osteoporosis fracture (*n* = 5130) with genetic risk score, BMD, and other risk factors as predictors by using ML methods such as RF, NN, gradient boosting, and LR. Unsurprisingly, performance of LR was significantly worse than the more advanced techniques of of RF, NN, gradient boosting. However, in contrast to previous studies, recent research has shown that traditional AI methods may still hold promise for fracture risk prediction. For example, when de Vries et al. [43] developed Cox regression, random survival forests (RSF) and an artificial neural network (ANN)-DeepSurv model to predict the risk of a future MOF, Cox regression outperformed RSF (*p* = 0.043 and *p* = 0.023) and ANN-DeepSurv (*p* = 0.043) with a C-index of 0.625 (0.562–0.689), pulling back a game for traditional AI methods These results suggest that the adage "new is good" may not always hold true in the field of AI, and reinforce the importance of rigorous comparative studies to identify the most effective methods for specific clinical applications.

In general, the development of AI algorithm has led the prediction of osteoporotic fracture into a new aera. Compared to traditional predictive tools, AI algorithms have achieved superior efficiency in fracture risk assessment. However, among the various types of AI algorithms currently available, no single algorithm has demonstrated consistently outstanding predictive performance. Additionally, issues related to universality and practicality still need to be addressed, highlighting the need for continued improvement in this field. To achieve optimal results, it is necessary to develop specific AI algorithms tailored to the needs of distinct populations, such as diabetic patients, which would enable the creation of specialized predictive tools for these high-risk groups.

## Efficacy of AI in predicting osteoporotic fracture in diabetes population

As previously discussed, conventional fracture risk prediction tools have shown limited efficacy when applied to osteoporosis patients with diabetes. Now what about the performance of AI algorithm? 20 literature results were identified in PubMed using the search terms ("Fracture"[All Fields] OR "Fragility Fracture"[All Fields] OR "Osteoporotic Fracture"[All Fields]) AND ("Artificial Intelligence"[All Fields] OR "Deep Learning"[All Fields] OR "Machine Learning"[All Fields]) AND ("Diabetes"[All Fields] OR "Hyperglycemia"[All Fields] OR "Abnormal glucose metabolism"[All Fields]), and 23 literature results were identified in Web of Science with search terms (((TS = (Artificial Intelligence OR Deep Learning OR Machine Learning)) AND TS = (Fracture OR Fragility Fracture OR Osteoporotic Fracture)) AND TS = (Diabetes OR Hyperglycemia OR Abnormal glucose metabolism)). However, only 3 records were accurately related to application of AI on fracture risk prediction in diabetes patients after review of titles and abstracts.

In 2022, Chen et al. [40] developed a hybrid model combining XGBoost with deep neural network (DNN) to predict the fracture risk of patients using data of 14,419 diabetes patients. Various machine learning methods were used simultaneously in this study, including LR, RF, kNN, Support Vector Machine (SVM), Decision Tree (DT), Extremely Randomized Trees (ERT), Gradient Boosting Decision Tree (GBDT), AdaBoost, CatBoost, XGBoost, and multilayer perceptron (MLP, a DNN derived algorism). Accuracy of the ML methods ranged from 67.76% to 86.08%, precision ranged from 70.41% to 87.69%. LR presented the worst performance (accuracy of 67.76% and precision of 70.41%), while XGboost (accuracy of 86.08% and precision of 87.69%) and MLP (accuracy of 82.78% and precision of 84.18%). Furthermore, the authors combined the best two methods to develop a new one and achieved the best performance (accuracy of 90.38%, precision of 90.52%, and AUC of approximately 0.90). Under the premise that the fracture prediction ability of traditional tools in diabetes patients is greatly reduced, it is encouraging that AI shows such excellent prediction efficiency. In the following year, two exciting studies emerged. Chu et al. [57] developed Probabilistic Classification Vector Machines (PCVM) algorithm to construct risk prediction models for fractures apart from the 6 algorithms LR, SVM, RF, DT, GBDT, XGBoost, which were conducted in the research above. What is delightful is that PCVM achieved the best f1 scores (0.97), surpassing LR (0.75), SVM (0.83), RF (0.84), DT (0.85), GBDT (0.87), XGBoost (0.88). Research of Chen et al. [40] determined 18 influencing factors of fracture risks of patients with diabetes while Chu et al. [57] determined 17 influencing factors, these influencing factors were easy to obtain and do not require precise inspection. To predict the risk of hip fractures in a more accurate way, Yosibash et al. [58] developed a ML algorithm with autonomous finite element analyses (AFE) based on CT scans for hip fracture risk assessment in type 2 diabetic mellitus (T2DM). The research results showed a sensitivity of 92% and specificity of 88% (cross-validation area under the curve [AUC] 0.92) among T2DM patients, indicating that AL algorithm has the potential to showcase more advantages in the accuracy of fracture risk assessment for diabetes patient combining with the advancement of imaging technique.

Current evidences suggest that Denosumab, Ibandronate, and Teriparatide are considered the most successful drugs for postmenopausal osteoporosis-related fragility fractures [59, 60]. Risedronate, alendronate, zoledronate, denosumab, or etidronate have also shown good efficacy in preventing fractures in corticosteroid-induced osteoporosis (CIO) [61]. The effectiveness of osteoporosis treatment is believed to be related to factors such as polymorphisms of the vitamin D receptor (VDR) [62], and biochemical markers of bone turnover (BTMs), such as the bone alkaline phosphatase (bALP), procollagen type I N propeptide (PINP), serum cross-linked C-telopeptides of type I collagen (bCTx), and urinary cross-linked N-telopeptides of type I collagen (NTx) [63, 64]. However, there is a lack of large-scale clinical studies on osteoporosis patients with diabetes, which is related to the lack of prediction methods for osteoporotic fractures in patients with osteoporosis and diabetes, which leads to the lack of awareness of this aspect. Although research in this field is still limited, it is noteworthy that various machine learning methods were employed in these researches, leveraging large dataset for training purposes. As such, these findings lend credibility to the potential utility of AI-driven approaches for improving risk prediction in diabetic patients. Nevertheless, further research is warranted to validate and extend these results, as well as to explore the broader applicability of AI in addressing clinical challenges related to osteoporosis management in high-risk populations.

## Conclusion

In the domain of osteoporotic fracture prediction, there remains a substantial need for improved performance of traditional tools such as FRAX, QFracture, and Garvan FRC when applied to patients with diabetes. Under such conditions, AI algorithm holds a bright future in enhancing the accuracy of fracture risk prediction in osteoporosis patients with diabetes. Notably, advanced AI techniques are rapidly evolving, while conventional methods such as linear regression continue to demonstrate utility. However, the limitations of AI methods comprising the demand of large amount of training data, inconvenience for clinical application, and unpredictable universality of the model. To address these challenges, it is urgent to develop new machine learning models using large, real-world data sets from patients with diabetes and osteoporosis exhibiting regional or national characteristics. By leveraging these approaches, it may be possible to establish a novel, widely accepted osteoporotic fracture prediction tool that can better serve the needs of high-risk patient populations.
